# Supplementary material for: Marine phytoplankton functional types exhibit diverse responses to thermal change
Source: Nat Commun. 2021 Nov 5;12:6413. doi: 10.1038/s41467-021-26651-8 (PMC8571312; doi:10.1038/s41467-021-26651-8)
Supplement: Supplementary file 1 — Supplementary Information [file 41467_2021_26651_MOESM1_ESM.pdf]

Supplementary Information for

Marine Phytoplankton Functional Types Exhibit Diverse Responses to Thermal Change

S.I. Anderson, A.D. Barton, S. Clayton, S. Dutkiewicz, and T.A. Rynearson
Correspondence to: sianderson@uri.edu (S.I.A.); rynearson@uri.edu (T.A.R.)

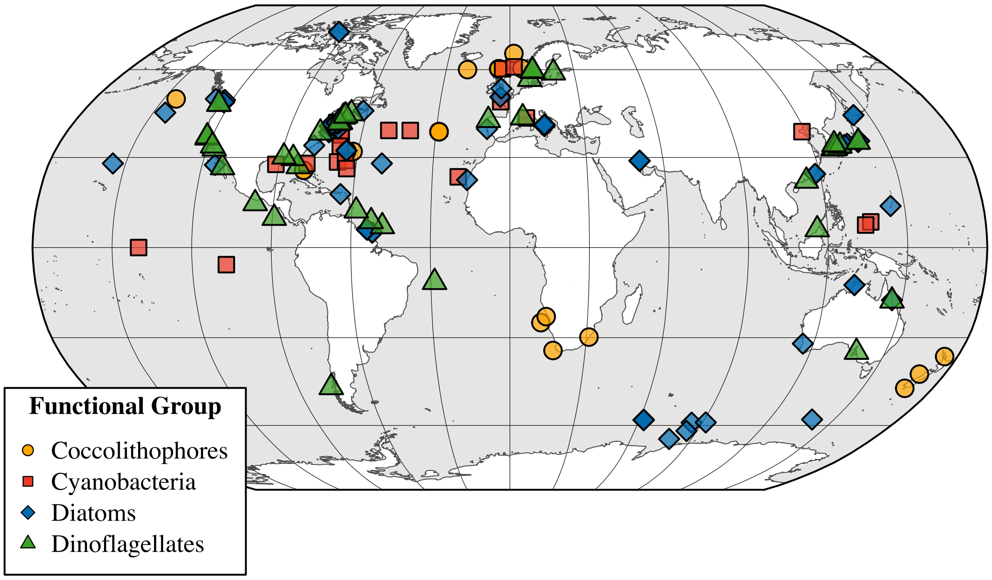

Supplementary Fig. 1. Isolation locations of strains of known origin. Colors and shape characterize the functional group to which each isolate belongs.

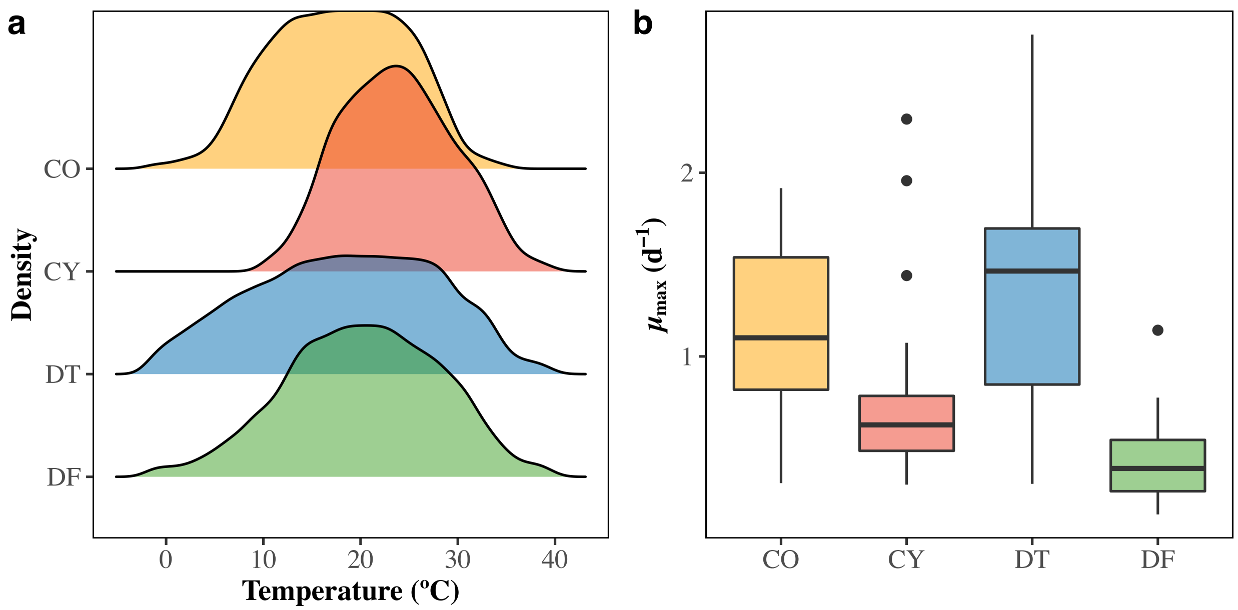

Supplementary Fig. 2. Thermal range and maximum growth for each functional type. (**a**) Density of viable strains from each functional group (Coccolithophores = CO, cyanobacteria = CY, diatoms = DT, dinoflagellates = DF). Strains were considered viable at a given temperature if their growth rates were at least 20% of their μ_{max}. (**b**) Growth maxima (μ_{max}) distribution for each functional group. Box plots extend from the first to the third quartile, with a line denoting the median, whiskers extending to the greatest value within 1.5x interquartile range, and points displaying data outliers. Only strains for which reaction norms could be fit were used in calculations (CO = 30, CY = 32, DT = 135, DF = 46). Of the groups examined, cyanobacteria displayed the most narrowly distributed reaction norms along the temperature gradient (**a**, Supplementary Table 1), while dinoflagellates exhibited lower (Supplementary Table 1) and less variable μ_{max} than all other groups examined (**b**).

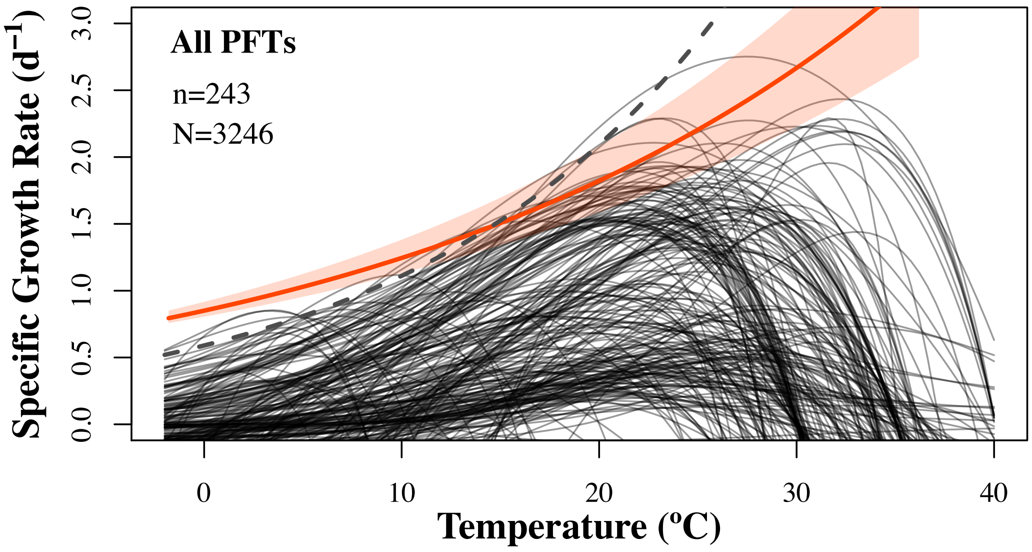

Supplementary Fig. 3. Thermal dependency of all PFTs. Exponential curve (colored line) fit to all measured growth rates (*N*) using a 99th quantile regression and compared to the that of Eppley[1] (grey dashed line). The 95% confidence interval (shading) was determined using Markov chain marginal bootstrapping[2] and are centered at the median. Together, PFTs are characterized by a Q_{10} of 1.46. Thermal reaction norms (*n)* for each isolate characterized are shown in black.

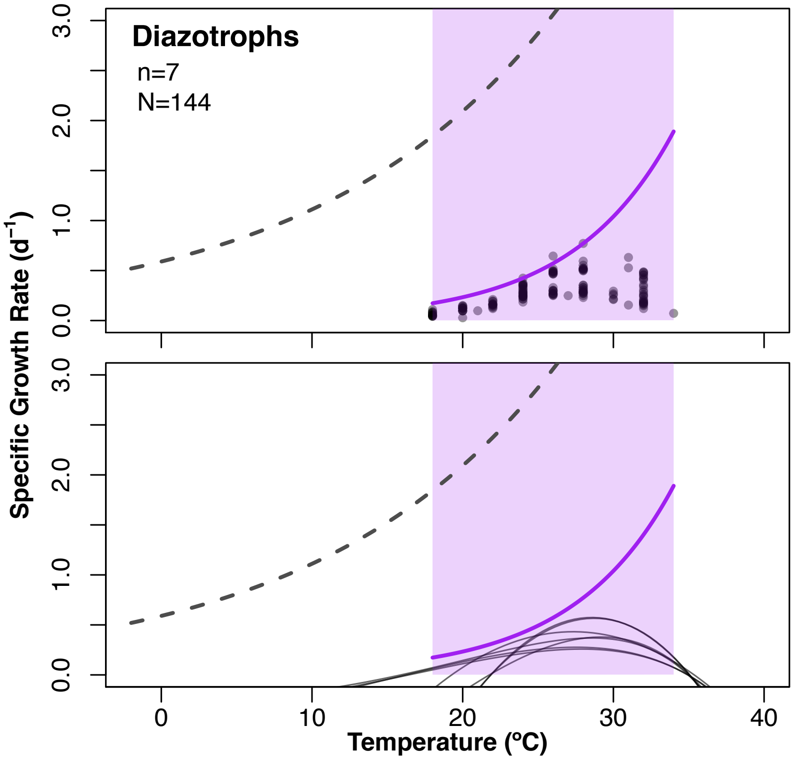

Supplementary Fig. 4. Diazotroph thermal reaction norms. Exponential curve (purple line) was fit to measured growth rates (*N,* top panel) using a 99th quantile regression and compared to the widely-used Eppley curve[5], which assessed phytoplankton collectively (grey dashed line). Extent of curve denotes limits of data. 95% confidence intervals (purple shading) were determined using Markov chain marginal bootstrapping[65] and are centered at the median. Thermal reaction norms (*n)* for each isolate characterized are shown in black (bottom panel). Data was deemed insufficient to carry out subsequent analyses.

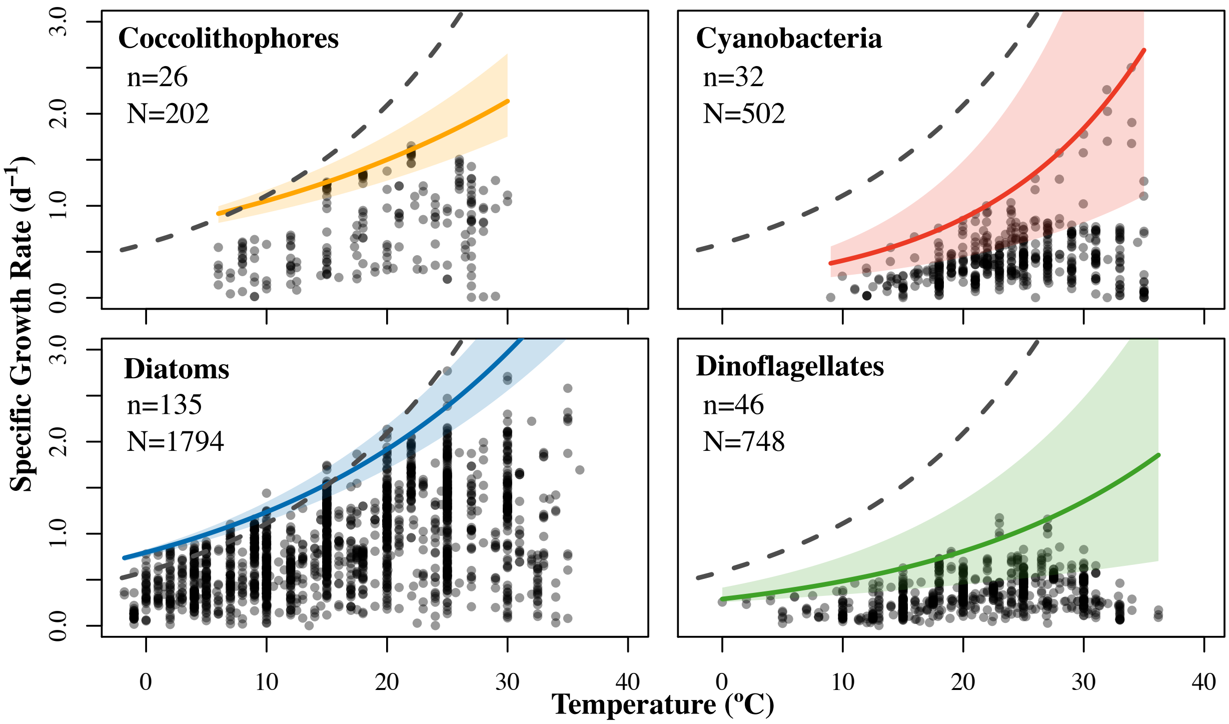

Supplementary Fig. 5. Temperature dependencies for each PFT. Exponential curves from this study (colored lines) were fit to measured growth rates (*N*, points) using a 99th quantile regression and compared to the Eppley curve[1] (grey dashed line). Confidence intervals (shading) were determined using Markov chain marginal bootstrapping[2] and are centered at the median. The number of individuals examined in each functional group is given by *n*.

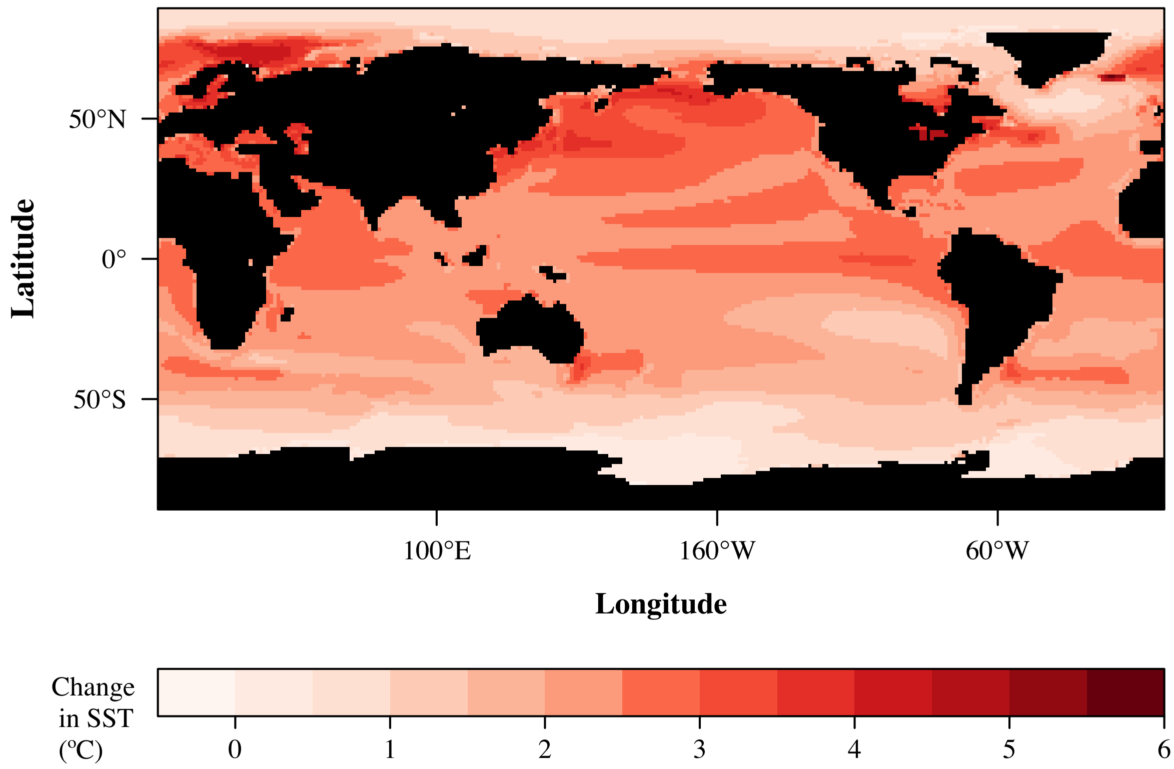

Supplementary Fig. 6. Earth System model projection of sea surface temperature change (ºC) between 1950-1970 and 2080-2100 under the RCP 8.5 climate scenario.

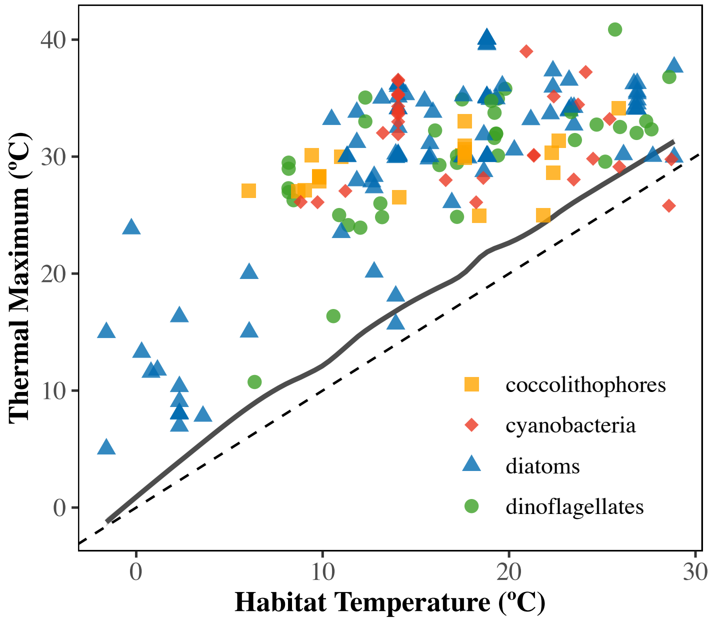

Supplementary Fig. 7. Correlation between thermal maxima and habitat temperature at site of strain origin. Dashed line indicates 1:1 relationship, while solid line depicts expected warming at each habitat location under the RCP 8.5 climate scenario. Only strains of known origin are shown (coccolithophores = 26, cyanobacteria = 31, diatoms = 123, dinoflagellates = 46).

Supplementary Table 1. Sources of phytoplankton growth rates added to original Thomas et al.[3] compilation. Listed are the number of strains (*n*) added from each study, as well as the phytoplankton functional type (PFT) from which they derive.

**Source**	**n**	**PFT**
Anderson and Rynearson 2020 [4]	19	Diatoms
Aranguren-Gassis et al. 2019 [5]	1	Diatoms
Baker 2018 [6]	1	Dinoflagellates
Boyd et al. 2019 [7]	4	Diatoms
Kling et al. 2020 [8]	11	Cyanobacteria
Mackey et al. 2013 [9]	3	Cyanobacteria
Pittera et al. 2014 [10]	6	Cyanobacteria
Stawiarski et al. 2016 [11]	3	Cyanobacteria
Zhang et al. 2014 [12]	11	Coccolithophores

Supplementary Table 2. Results from Dunn's multiple comparison test of PFT thermal traits. Pair-wise comparisons were carried out between PFTs of strain-specific niche widths and maximum growth rates (μ_{max}). Niche width represents the range of temperatures where strains exceed 20% of their μ_{max}. The z-test statistic is given by *Z*, two-sided adjusted p-values are given by *p*, and asterisks denote significantly different thermal traits among PFT strains ($\alpha = 0.05$).

Trait	PFT Comparison	Z	p
niche width	coccolithophores - cyanobacteria	3.40	0.0020*
	coccolithophores - diatoms	-3.26	0.0022*
	cyanobacteria - diatoms	-7.73	<0.0001*
	coccolithophores - dinoflagellates	0.08	0.9375
	cyanobacteria - dinoflagellates	-3.67	0.0010*
	diatoms - dinoflagellates	3.96	0.0004*
μ_{max}	coccolithophores - cyanobacteria	2.72	0.0132*
	coccolithophores - diatoms	-1.37	0.1694
	cyanobacteria - diatoms	-4.92	<0.0001*
	coccolithophores - dinoflagellates	5.78	<0.0001*
	cyanobacteria - dinoflagellates	2.89	0.0116*
	diatoms - dinoflagellates	9.57	<0.0001*

Supplementary Table 3. Functional group thermal equation coefficients. Coefficients for μ_{max}-temperature exponential relationships characterized using each functional group's discrete thermal growth measurements (*N*). The y-intercept is given by *a*, and *b* characterizes the rate at which μ_{max} scales with temperature. The 95% confidence intervals (CI) for each parameter were discerned using a Markov chain marginal bootstrap over 10,000 iterations.

**Functional group**	**N**	**a**	**95% CI for a**	**b**	**95% CI for b**
All	3246	0.850	[0.806, 0.919]	0.038	[0.034, 0.041]
Coccolithophores	202	0.741	[0.672, 0.779]	0.035	[0.032, 0.041]
Cyanobacteria	502	0.190	[0.130, 0.251]	0.076	[0.061, 0.090]
Diatoms	1794	0.798	[0.759, 0.832]	0.044	[0.040, 0.048]
Dinoflagellates	748	0.291	[0.257, 0.418]	0.051	[0.027, 0.060]

Supplementary Table 4. AICc model comparison of quantile regression analyses. Results from an Akaike information criterion with correction for small sample size (AICc) model comparison. A model incorporating a PFT-temperature interaction (Group*Temperature) was compared with one that treated factors independently (Group + Temperature) to discern whether PFT thermal dependencies should be evaluated separately. The best model (lowest AICc score) incorporated a PFT-temperature interaction, supporting our decision to fit 99th quantile regressions to each PFT's growth data separately. Degrees of freedom (df) for each assessed model are listed.

**Model**	**AICc**	**df**
Group*Temperature	0.0	8
Group + Temperature	187.5	5

Supplementary Table 5. Exponential curve fitting methods employed to assess phytoplankton thermal dependencies. The temperature to maximum growth (μ_{max}) relationship has been assessed using several methodologies. Here, asterisks denote studies that assessed phytoplankton as a whole, either omitting PFT taxonomic designations or treating them as an independent variable in a mixed effect model. The work by Eppley[1] has been intentionally excluded, as it utilized a visual assessment of the μ_{max}.

**Method**	**Study**
99th quantile regression	Bissinger et al. 2008[13]*<br>Stawiarski et al. 2016[11]<br>Kremer et al. 2017[14]*<br>This study
Ordinary least squares regression	Buitenhuis et al. 2008[15]<br>Chen et al. 2017[16]
Metabolic Theory of Ecology	Kremer et al. 2017[14]*

References:

1. Eppley, R. W. Temperature and phytoplankton growth in the sea. *Fish. Bull.* **70**, 1063–1085 (1972).
2. He, X. & Hu, F. Markov chain marginal bootstrap. *J. Am. Stat. Assoc.* **97**, 783–795 (2002).
3. Thomas, M. K., Kremer, C. T., Klausmeier, C. A. & Litchman, E. A global pattern of thermal adaptation in marine phytoplankton. *Science* **338**, 1085–1088 (2012).
4. Anderson, S. I. & Rynearson, T. A. Variability Approaching the Thermal Limits Can Drive Diatom Community Dynamics. *Limnol. Oceanogr.* **65**, 1961–1973 (2020).
5. Aranguren-Gassis, M., Kremer, C. T., Klausmeier, C. A. & Litchman, E. Nitrogen limitation inhibits marine diatom adaptation to high temperatures. *Ecol. Lett.* **22**, 1860–1869 (2019).
6. Baker, K. G. *et al.* Thermal niche evolution of functional traits in a tropical marine phototroph. *J. Phycol.* **54**, 799–810 (2018).
7. Boyd, P. W. Physiology and iron modulate diverse responses of diatoms to a warming Southern Ocean. *Nat. Clim. Chang.* **9**, 148–152 (2019).
8. Kling, J. D. *et al.* Dual thermal ecotypes detected within a nearly genetically-identical population of the unicellular marine cyanobacterium Synechococcus. *bioRxiv* (2020). doi:10.1101/2020.05.27.119842
9. Mackey, K. R. M. *et al.* Effect of Temperature on Photosynthesis and Growth in Marine Synechococcus spp. *Plant Physiol.* **163**, 815–829 (2013).
10. Pittera, J. *et al.* Connecting thermal physiology and latitudinal niche partitioning in marine Synechococcus. *ISME J.* **8**, 1221–1236 (2014).
11. Stawiarski, B., Buitenhuis, E. T. & Quéré, C. Le. The physiological response of

picophytoplankton to temperature and its model representation. *Front. Mar. Sci.* **3**, 1–13 (2016).

12. Zhang, Y. *et al.* Between- and within-population variations in thermal reaction norms of the coccolithophore Emiliania huxleyi. *Limnol. Oceanogr.* **59**, 1570–1580 (2014).
13. Bissinger, J. E., Montagnes, D. J. S., Sharples, J. & Atkinson, D. Predicting marine phytoplankton maximum growth rates from temperature: Improving on the Eppley curve using quantile regression. *Limnol. Oceanogr.* **53**, 487–493 (2008).
14. Kremer, C. T., Thomas, M. K. & Litchman, E. Temperature- and size-scaling of phytoplankton population growth rates: Reconciling the Eppley curve and the metabolic theory of ecology. *Limnol. Oceanogr.* **62**, 1658–1670 (2017).
15. Buitenhuis, E. T., Pangerc, T., Franklin, D. J., Quéré, C. Le & Malin, G. Growth rates of six coccolithophorid strains as a function of temperature. *Limnol. Oceanogr.* **53**, 1181–1185 (2008).
16. Chen, B. & Laws, E. A. Is there a difference of temperature sensitivity between marine phytoplankton and heterotrophs? *Limnol. Oceanogr.* **62**, 806–817 (2017).
