# Supplementary material for: Marine phytoplankton functional types exhibit diverse responses to thermal change
Source: Nat Commun. 2021 Nov 5;12:6413. doi: 10.1038/s41467-021-26651-8 (PMC8571312; doi:10.1038/s41467-021-26651-8)
Supplement: Supplementary file 3 — Reporting Summary [file 41467_2021_26651_MOESM3_ESM.pdf]

nature portfolio

Corresponding author(s): S.I.Anderson (sianderson@uri.edu) & T.A.Rynearson (rynearson@uri.edu)

Last updated by author(s): Sep 14, 2021

# Reporting Summary

Nature Portfolio wishes to improve the reproducibility of the work that we publish. This form provides structure for consistency and transparency in reporting. For further information on Nature Portfolio policies, see our Editorial Policies and the Editorial Policy Checklist.

## Statistics

For all statistical analyses, confirm that the following items are present in the figure legend, table legend, main text, or Methods section.

| n/a | Confirmed | |
|---|---|---|
| ☐ | ☒ | The exact sample size (*n*) for each experimental group/condition, given as a discrete number and unit of measurement |
| ☐ | ☒ | A statement on whether measurements were taken from distinct samples or whether the same sample was measured repeatedly |
| ☐ | ☒ | The statistical test(s) used AND whether they are one- or two-sided<br>*Only common tests should be described solely by name; describe more complex techniques in the Methods section.* |
| ☐ | ☒ | A description of all covariates tested |
| ☐ | ☒ | A description of any assumptions or corrections, such as tests of normality and adjustment for multiple comparisons |
| ☐ | ☒ | A full description of the statistical parameters including central tendency (e.g. means) or other basic estimates (e.g. regression coefficient) AND variation (e.g. standard deviation) or associated estimates of uncertainty (e.g. confidence intervals) |
| ☐ | ☒ | For null hypothesis testing, the test statistic (e.g. *F*, *t*, *r*) with confidence intervals, effect sizes, degrees of freedom and *P* value noted<br>*Give P values as exact values whenever suitable.* |
| ☐ | ☒ | For Bayesian analysis, information on the choice of priors and Markov chain Monte Carlo settings |
| ☐ | ☒ | For hierarchical and complex designs, identification of the appropriate level for tests and full reporting of outcomes |
| ☐ | ☒ | Estimates of effect sizes (e.g. Cohen's *d*, Pearson's *r*), indicating how they were calculated |

*Our web collection on statistics for biologists contains articles on many of the points above.*

## Software and code

Policy information about availability of computer code

| | |
|---|---|
| Data collection | Data was compiled from the literature. When growth data was not available in spreadsheet form, GraphClick software (version 3.0.3) was employed to digitize rate measurements. |
| Data analysis | We used the statistical program R 4.0.2 to analyze the data in this study. Quantile regressions were estimated using the quantreg package (version 5.86) and maximum likelihood estimations were conducted using the bbmle package (version 1.0.24). Scripts for data analyses have been made available in a GitHub repository (github.com/sianderson/PFT_thermal_response) and archived at Zenodo (https://doi.org/10.5281/zenodo.5507532). |

For manuscripts utilizing custom algorithms or software that are central to the research but not yet described in published literature, software must be made available to editors and reviewers. We strongly encourage code deposition in a community repository (e.g. GitHub). See the Nature Portfolio guidelines for submitting code & software for further information.

## Data

Policy information about availability of data

All manuscripts must include a data availability statement. This statement should provide the following information, where applicable:

- Accession codes, unique identifiers, or web links for publicly available datasets
- A description of any restrictions on data availability
- For clinical datasets or third party data, please ensure that the statement adheres to our policy

Growth measurements (doi:10.26008/1912/bco-dmo.839696.1), thermal capacities (doi:10.26008/1912/bco-dmo.839713.1), and estimated thermal traits (doi:10.26008/1912/bco-dmo.839689.1) have been made available through the Biological and Chemical Ocean Data Management Office (BCO-DMO).

# Field-specific reporting

Please select the one below that is the best fit for your research. If you are not sure, read the appropriate sections before making your selection.

☐ Life sciences ☐ Behavioural & social sciences ☒ Ecological, evolutionary & environmental sciences

For a reference copy of the document with all sections, see nature.com/documents/nr-reporting-summary-flat.pdf

# Ecological, evolutionary & environmental sciences study design

All studies must disclose on these points even when the disclosure is negative.

| | |
|---|---|
| Study description | We conducted a meta-analysis of thermal response data to characterize key phytoplankton functional types (PFTs) and their relative abilities to cope with ocean warming. This included assessing each PFTs thermal dependency (Q10) and static capacity for thermal change. The PFTs we evaluated included four principal contributors to marine productivity: diatoms (strains (n)=135), dinoflagellates (n=46), coccolithophores (n=30), and cyanobacteria (n=32). We then employed sea surface temperature projections from an ensemble of Earth System Models to assess how PFT growth and geographical range may be altered in a future ocean. |
| Research sample | Beginning with a growth rate compilation by Thomas et al. (2012), we added data from nine studies published after 2012 (Anderson and Rynearson, 2020; Aranguren-Gassis et al., 2019; Baker, 2018; Boyd et al., 2019; Kling et al., 2020; Mackey et al., 2013; Pittera et al., 2014; Stawiarski et al., 2016; Zhang et al., 2014). This resulted in a compilation of thermal growth rates from four functional groups, which comprised 243 strains and 3,246 discrete growth rate measurements from a broad range of temperatures and locations. In total, our dataset contained the following growth rate measurements (N): coccolithophores (N=202), cyanobacteria (N=502), diatoms (N=1794), and dinoflagellates (N=748). |
| Sampling strategy | Analyses for this study were conducted using data from the literature. We used 99th quantile regressions, according to the method outlined in Bissinger et al. (2008), to determine whether the sample size from each phytoplankton functional type was sufficient; both in terms of the number and thermal range of thermal growth rate measurements. |
| Data collection | This study does not include new experimental findings, as data was compiled from the literature. When growth data was not made available in spreadsheet form, GraphClick software (version 3.0.3) was employed to digitize rate measurements from published figures. An ensemble mean of modeled sea surface temperatures from two 20-year time frames (1950-1970; 2080-2100) were utilized from the Coupled Model Intercomparison Project phase 5 (CMIP5). |
| Timing and spatial scale | Thermal growth rates were compiled from literature published from 1935 to 2020. They include phytoplankton collected globally, but are restricted to marine or estuarine sources. |
| Data exclusions | We focused our efforts on marine phytoplankton, excluding freshwater species which have evolved under physical dynamics that strongly differ from those of marine environments. Thermal growth rates followed the selection criteria outlined in Thomas et al. (2012), with a few modifications. We broadened our criteria to include growth rates measured at greater than 80 µmol photons m2s-1 when day length equaled 24 hours, allowing for the inclusion of more cyanobacteria. We eliminated studies which exposed strains to fluctuating nutrient concentrations, as there was concern about the comparability of the resulting reaction norms. Additionally, the cyanobacteria group was constrained to eliminate diazotrophic species, which are characterized by fundamentally different physiological processes, which could impact group characterizations. Though diazotrophs are significant ecological contributors, the data available was deemed insufficient to characterize them independently (see Sampling strategy). Additionally, dinoflagellate growth rates were verified to be autotrophically obtained (strains grown on medium only). |
| Reproducibility | R code and growth data have been made available so analyses can be reproduced. |
| Randomization | Phytoplankton functional groups were assigned according to information from the original studies. Data was not otherwise grouped. |
| Blinding | Data for this study was collected from the literature prior to analysis and growth data can be traced back to its original sources. |

Did the study involve field work? ☐ Yes ☒ No

# Reporting for specific materials, systems and methods

We require information from authors about some types of materials, experimental systems and methods used in many studies. Here, indicate whether each material, system or method listed is relevant to your study. If you are not sure if a list item applies to your research, read the appropriate section before selecting a response.

## Materials & experimental systems

| n/a | Involved in the study |
| --- | --- |
| x | Antibodies |
| x | Eukaryotic cell lines |
| x | Palaeontology and archaeology |
| x | Animals and other organisms |
| x | Human research participants |
| x | Clinical data |
| x | Dual use research of concern |

## Methods

| n/a | Involved in the study |
| --- | --- |
| x | ChIP-seq |
| x | Flow cytometry |
| x | MRI-based neuroimaging |
